# Artificial intelligence and transcatheter aortic valve implantation-induced conduction disturbances—adding insight beyond the human ‘I’

**DOI:** 10.1093/ehjdh/ztae040

**Published:** 2024-06-03

**Authors:** Patrick Houthuizen, Peter P T de Jaegere

**Affiliations:** Department of Cardiology, Catharina Hospital, Michelangelolaan 2, 5623 EJ Eindhoven, the Netherlands; Department of Cardiology, Thoraxcenter, Erasmus Medical Center, ‘s-Gravendijkwal 230, 3015 CE Rotterdam, the Netherlands

## Introduction

Mrs. Smith, an active 83-year-old female with a medical history encompassing hypertension and type 2 diabetes mellitus, presents with symptomatic severe aortic valve stenosis. Her left ventricular function is normal, and there is no evidence of obstructive coronary artery disease. She undergoes a transcatheter aortic valve implantation (TAVI) with a 34 mm self-expandable prosthesis. According to the interventional cardiologist, the procedure was uncomplicated, thereby considering the post-operative left bundle branch block (LBBB) as trivial. As a 5-day post-operative continuous rhythm monitoring reveals no other conduction abnormality or delay, Mrs. Smith is discharged home with a persistent LBBB. However, 2 weeks later, she is re-admitted due to a cardiac syncope attributed to high-grade atrioventricular block (HAVB), necessitating permanent pacemaker implantation (PPMI).

## Discussion

The clinical scenario above will be well appreciated by every clinician involved in the care of patients with TAVI. Although TAVI is the recommended intervention over surgical aortic valve replacement (SAVR) in the elderly population, given its less invasive nature,^1^ both post-operative conduction disturbances and the necessity for PPMI remain a concern. For LBBB, in particular, it has been proven to negatively impact outcome and survival.^2,3^ As indications for TAVI tend to shift to more younger patients and/or lower risk of SAVR, it is even more important to identify patients who are at risk for the development of conduction disturbances.

In this issue of the European Heart Journal—Digital Health, Jia and Li *et al*. propose an interesting solution to do so based on the pre-operative ECG.^4^ They developed an artificial intelligence (AI) model for ECG analysis to overcome the problem that human ECG interpretation is hampered by subjectiveness and level of expertise. This adds up to the ongoing discussion about the ECG criteria of LBBB and, hence, strikingly demonstrates the limitations of human ECG reading, interpretation, and classification of abnormalities.^5,6^

The base of their solution is elegantly simple, as the authors employed a convolutional neural network (CNN) capable of processing ECG images instead of raw data signals. Although their AI model is based on a relatively small population of 718 patients and 1354 ECGs, the performance is good with an area under the curve 0.76, and this did not significantly changed to 0.78 when adding clinical data into the model. Interestingly, and less emphasized by the authors, is that incorporating clinical and procedural variables decreased sensitivity from 0.88 to 0.79, while specificity increased from 0.62 to 0.75, with corresponding changes in the positive and negative predictive values—measures that hold more clinical relevance. Thus, this AI analysis, solely based on the pre-operative ECG, has the potential to be further developed into a risk stratification tool to aid the clinician in deciding which procedure and valve are best for the individual patient.

Although the authors need to be complimented for this original concept, there are some drawbacks that merit discussion.

At first glance, employing ECG images as an input variable appears appealing, given its ease to implement in clinical practice as it is not limited to the format of the (often vendor-locked) native data. Nevertheless, the input of the algorithm is an ECG image that is routinely used in clinical practice, which is typically a PDF format intended for human interpretation. Multiple filters are applied to reduce signals that are considered ‘noise’. However, it is worth noting that the raw signal might contain important information for the AI algorithm that is not appreciated by the human eye.

Furthermore, the chosen primary endpoint of conduction disturbances up to 30 days post-operative, defined as PPMI, HAVB, and TAVI-induced LBBB as adjudicated by heart team members using the ECG made at the hospital where patients were seen for follow-up. As it is known that LBBB resolves over time in up to 40% of patients,^7^ the authors decided to only regard a persistent LBBB as an endpoint. This seems a plausible choice at first sight given the fact that only persistent LBBB has prognostic implications; however, this holds the presumption that the model is capable of distinguishing between a temporary and a persistent LBBB. This underscores the fact that while learning techniques such as CNNs are highly valuable in identifying associations within large multidimensional datasets, they do not offer insight into the underlying pathophysiology of the event.

On the contrary, for PPMI, the authors included any pacemaker implantation post-operative as an endpoint, although conduction disorders in the acute phase can be temporary as well. Indeed, on follow-up, a considerable number of patients with a PPMI post-TAVI exhibit no pacing at all.^8^ Of note, the main component of the composite endpoint was PPMI with a prevalence of approximately 24%, while the others were seen in about 1–4% of patients. Notwithstanding criteria for PPMI, it remains an endpoint that is surrounded by bias.

Further, as for the clinical data in this study, a self-expandable valve was implanted in more than 90% of procedures. This renders the model less reliable for balloon-expandable valves, which carry a significantly lower risk for conduction abnormalities. It should be further noted that some clinical valuable input variables with a sound pathophysiologic basis were not use in the model (i.e. calcium load, length of membranous septum length, valve/annulus ratio, and depth of implantation).^9,10^

Nevertheless, this study adds to the growing evidence that AI improves insight in clinical prognosis. It is an important step to further enhance risk stratification and patient selection for TAVI in order to reduce the occurrence of conduction disorders, which still remain a frequent and important complication. Besides further refinement of the model, the next step should include the possibility to distinguish ‘malignant’ from ‘non-malignant’ LBBB, identifying patients with post-operative conduction disorders that need close monitoring. Not by the human eye, but with the fast-expanding possibilities of the A-‘eye’.

## Data Availability

No new data were generated or analysed in support of this research.
